# Supplementary material for: Gender Differences in Appropriate Shocks and Mortality among Patients with Primary Prophylactic Implantable Cardioverter-Defibrillators: Systematic Review and Meta-Analysis
Source: PLoS One. 2016 Sep 12;11(9):e0162756. doi: 10.1371/journal.pone.0162756 (PMC5019464; doi:10.1371/journal.pone.0162756)
Supplement: S1 Table — (DOCX) [file pone.0162756.s002.docx]

**Supporting information Table S1**

Sensitivity analyses for all three endpoints removing one study at a time (confidence intervals (and p-values) obtained with Knapp-Hartung correction)

A) Appropriate shock.

| Study left out | HR | CI 95% | p-value | Tau | p-value* |
| --- | --- | --- | --- | --- | --- |
| van der Heijden et al. | 0.58 | 0.37-0.88 | 0.0231 | 0.18 | 0.263 |
| Seegers et al. | 0.62 | 0.38-1.00 | 0.0501 | 0.27 | 0.172 |
| Weeke et al. | 0.69 | 0.52-0.92 | 0.0222 | 0 | 0.871 |
| Wijers et al. | 0.60 | 0.38-0.97 | 0.0412 | 0.27 | 0.149 |
| Gatzoulis et al. | 0.61 | 0.38-0.98 | 0.0435 | 0.27 | 0.150 |
| MacFadden et al. | 0.58 | 0.35-0.97 | 0.0410 | 0.25 | 0.159 |

B) All-cause mortality (multivariable models only)

| Study left out | HR | CI 95% | p-value | Tau | p-value* |
| --- | --- | --- | --- | --- | --- |
| van der Heijden et al. | 0.77 | 0.66-0.89 | 0.0018 | 0.13 | 0.055 |
| Seegers et al. | 0.75 | 0.65-0.87 | 0.0014 | 0.15 | 0.024 |
| Weeke et al. | 0.75 | 0.65-0.87 | 0.0013 | 0.15 | 0.022 |
| Wijers et al. | 0.77 | 0.68-0.87 | 0.0007 | 0.11 | 0.053 |
| Yung et al. | 0.74 | 0.64-0.86 | 0.0009 | 0.14 | 0.021 |
| Bilchick et al. | 0.72 | 0.63-0.84 | 0.0004 | 0.11 | 0.280 |
| Gigli et al. | 0.75 | 0.66-0.85 | 0.0003 | 0.10 | 0.035 |
| Hage et al. | 0.75 | 0.65-0.87 | 0.0012 | 0.15 | 0.022 |
| Masoudi et al. | 0.77 | 0.66-0.89 | 0.0024 | 0.13 | 0.114 |
| Providência et al. | 0.74 | 0.64-0.86 | 0.0007 | 0.13 | 0.022 |
| Rodríguez-Mañero et al. | 0.78 | 0.69-0.87 | 0.0004 | 0.09 | 0.077 |
| Smith et al. | 0.77 | 0.68-0.86 | 0.0003 | 0.09 | 0.052 |
| Amit et al. | 0.75 | 0.65-0.87 | 0.0010 | 0.14 | 0.021 |
| Gatzoulis et al. | 0.75 | 0.65-0.87 | 0.0010 | 0.14 | 0.021 |

C) Inappropriate shock.

| Study left out | HR | CI 95% | p-value | Tau | p-value* |
| --- | --- | --- | --- | --- | --- |
| MacFadden et al. | 1.06 | 0.05-22.8 | 0.8576 | 0.22 | 0.224 |
| van der Heijden et al. | 1.09 | 0.08-14.3 | 0.7532 | 0.10 | 0.297 |
| Weeke et al. | 0.94 | 0.16-5.43 | 0.7126 | 0 | 0.820 |

* P values for heterogeneity based on Q tests
