# Supplementary material for: Gender Differences in Appropriate Shocks and Mortality among Patients with Primary Prophylactic Implantable Cardioverter-Defibrillators: Systematic Review and Meta-Analysis
Source: PLoS One. 2016 Sep 12;11(9):e0162756. doi: 10.1371/journal.pone.0162756 (PMC5019464; doi:10.1371/journal.pone.0162756)
Supplement: S2 Table — (DOCX) [file pone.0162756.s003.docx]

**Supporting information Table S2**

Estimates for the cumulative incidence of death within the first 2 years after ICD implantation

|  |  | Death |  |
| --- | --- | --- | --- |
| van der Heijden et al |  | 0.11 (0.011) |  |
| Seegers et al* |  | 0.10 (0.012) |  |
| Weeke et al |  | 0.11 (0.012) |  |
| Wijers et al* |  | 0.09 (0.014) |  |
| Yung et al |  | 0.083 (0.008)° |  |
| Bilchick et al |  | -- |  |
| Gigli et al |  | 0.16 (0.036) |  |
| Hage et al |  | 0.12 (0.025) |  |
| Masoudi et al |  | -- |  |
| Providência et al. |  | -- |  |
| Rodríguez-Mañero et al. |  | 0.03 (0.015) |  |
| Smith et al |  | 0.03 (0.024) |  |
| Amit et al |  | -- |  |
| Gatzoulis et al |  | 0.09(0.022) |  |

Cumulative incidences were extracted from Kaplan-Meier curves in the individual publications. If only available for different subgroups, risk estimates were individually extracted and combined to the final estimate by weighting. Numbers within parentheses are estimates of standard deviations as estimated by the formula 0.5*sqrt(1/n).

* Results directly calculated from source data, including standard deviations

° From cumulative incidence of death in competing risks analysis (i.e. death without receiving an appropriate shock)
